# *Arabidopsis HECATE* genes function in phytohormone control during gynoecium development

**DOI:** 10.1242/dev.120444

**Published:** 2015-10-01

**Authors:** Christoph Schuster, Christophe Gaillochet, Jan U. Lohmann

**Affiliations:** Department of Stem Cell Biology, University of Heidelberg, Heidelberg D-69120, Germany

**Keywords:** Gynoecium development, HECATE, SPATULA, Phytohormones, Auxin, Cytokinin, *Arabidopsis*

## Abstract

The fruit, which develops from the fertilised gynoecium formed in the innermost whorl of the flower, is the reproductive organ and one of the most complex structures of an angiosperm plant. Phytohormones play important roles during flower and fruit patterning, morphogenesis and growth, and there is emerging evidence for a cross-talk between different classes of plant hormones throughout these processes. Here, we show that the bHLH transcription factors HECATE 1 (HEC1), HEC2 and HEC3, which have previously been identified as essential components of transmitting tract formation, affect both auxin and cytokinin responses during reproductive tissue development. We find that HEC1 interacts with SPATULA (SPT) to control carpel fusion and that both transcription factors restrict sensitivity to cytokinin in the gynoecium. In addition, HEC1 is tightly integrated into the auxin-signalling network at the levels of biosynthesis, transport and transcriptional response. Based on this data, we propose that HEC1 acts as a local modulator of auxin and cytokinin responses to control gynoecium development in *Arabidopsis*.

## Abstract (precis)

**Highlighted article:** In the developing reproductive tissue of plants, HECATE 1 and SPATULA coordinate auxin and cytokinin signalling to orchestrate the development of the gynoecium.

## INTRODUCTION

**Fig. 1.**
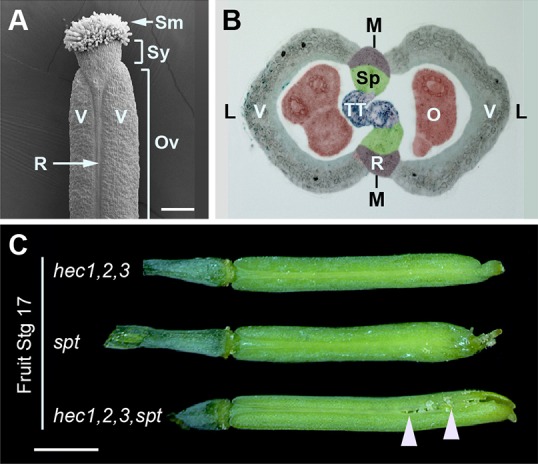
***HEC1* and *SPT* genetically interact to control gynoecium development.** (A,B) Longitudinal (A) and cross section view (B) of a wild-type *Arabidopsis* gynoecium. Stigma (Sm), style (Sy), ovary (Ov), valve (V), replum (R), septum (Sp), transmitting tract (TT), ovules (O), lateral (L) and medial (M) regions are shown. (C) Stage 17 fruits of *hec1,2,3*, *spt* and *hec1,2,3 spt* mutant plants. The apical part of *spt* fruits is unfused, and this phenotype is dramatically enhanced in *hec1,2,3 spt* quadruple mutant (arrowheads). Scale bars: 200 µm in A; 1 mm in C. See also supplementary material Fig. S1.

The flower is the defining structure of all angiosperms and has been studied in great detail in *Arabidopsis*. It consists of four types of organs: The outer, leaf-like sepals and petals, and the gametophyte-producing stamens and carpels. Flower development begins with the specification of floral meristem identity in a subgroup of cells at the flank of the shoot apical meristem (SAM). Later, flower primordia emerge, become separated from the main stem cell system of the shoot apical meristem and floral organ primordia arise. Fruits originate from the female reproductive organ, the gynoecium, which consists of two fused carpels. The gynoecium is capped by specialised epidermal cells called stigmata that function in pollen reception and neighbour the cylindrical style, which connects the apical stigma with the large central ovary. The outer layers of the ovary form the valves (carpel walls) and the replum (Fig. 1A,B). At pollination (flower stage 13), pollen grains germinate on the stigma and growing pollen tubes are guided through the transmitting tract to the ovules where fertilisation takes place (Edlund et al., 2004; Ferrándiz et al., 1999; Østergaard, 2009; Smyth et al., 1990).

Phytohormones play important roles in flower and fruit development. Auxin has been shown to act as a morphogen during gynoecium formation (Nemhauser et al., 2000). Several components of auxin biosynthesis, homeostasis and signalling are active in apical-basal fruit patterning, including the efflux facilitator PIN-FORMED 1 (PIN1), the protein kinase PINOID (PID), the auxin response factor ETTIN (ETT), and the RING-finger-like proteins STYLISH 1 (STY1) and STY2 (Bennett et al., 1995; Nemhauser et al., 2000; Okada et al., 1991; Sohlberg et al., 2006). STY1 promotes the production of auxin by inducing *YUCCA* (*YUC*) gene expression, and *sty1,2* double mutants can be partially rescued by exogenous auxin application (Cheng et al., 2006; Eklund et al., 2010; Ståldal et al., 2008). In addition, the specification of a local auxin minimum is crucial for the formation of the valve margin separation layer where fruit dehiscence takes place (Sorefan et al., 2009). *HECATE 1* (*HEC1*), *HEC2* and *HEC3* genes are involved in transmitting tract and stigma development and code for closely related basic helix-loop-helix (bHLH) transcription factors. Overexpression of any of these genes leads to pin-like phenotypes, and consequently it has been thought that they might coordinate auxin signalling in the gynoecium, but so far no direct evidence has been reported (Gremski et al., 2007). SPATULA (SPT), another bHLH transcription factor that controls carpel margin tissue development by promoting growth of the style, stigma and septum, interacts with INDEHISCENT (IND) to control *PID* expression (Alvarez and Smyth, 1999; Girin et al., 2011; Heisler et al., 2001). Interestingly, both *SPT* and *HEC* genes are under negative control of ETT, which prevents expression of these genes in abaxial regions during gynoecium development (Gremski et al., 2007; Heisler et al., 2001).

In addition to the established roles for auxin, it has been suggested that also cytokinin is important for gynoecium and fruit patterning, on the one hand by promoting proliferation at earlier stages of reproductive tract development, and on the other hand during valve margin morphogenesis at later stages (Marsch-Martínez et al., 2012a). Mutations in the *CYTOKININ OXIDASE/ DEHYDROGENASE 3* (*CKX3*) and *CKX5* genes, which catalyse the degradation of cytokinin, lead to increased gynoecium size and seed yields (Bartrina et al., 2011). Most interesting, there is emerging evidence that the balance between auxin and cytokinin, which has been shown to be essential in maintaining root and shoot stem cell systems, might also play a role during the development of the female reproductive tract (Jones et al., 2010; Marsch-Martínez et al., 2012b; Müller and Sheen, 2008; Zhao et al., 2010).

Recently, we showed that HEC1 coordinates the balance between proliferation and differentiation in the shoot apical meristem by promoting stem cell proliferation, while antagonising niche cell activity through physical interaction with SPT. In the SAM, HEC1 activates the expression of several type-A *ARABIDOPSIS RESPONSE REGULATOR* (*ARR*) genes and we proposed that these negative regulators of cytokinin signalling act as mobile signals to non-cell-autonomously interfere with the expression of the stem cell regulator WUSCHEL (Schuster et al., 2014).

Here, we investigate the function of HEC1 during reproductive tissue development. Our data reveals that HEC1, in the same way as in the SAM, interacts with SPT, and that both transcriptional regulators buffer cytokinin signals in the gynoecium. We also show that HEC1 controls auxin distribution during gynoecium development, via the direct regulation of *PIN1* and *PIN3* auxin transporters. This mechanism does not appear to be relevant for HEC activity in shoot stem cells, illustrating an exquisite spatial specificity. Together, our data highlight the conserved function of the interaction between HEC1 and SPT in modulating cytokinin signalling in diverse plant tissues, and suggests that both transcription factors might orchestrate the cross-talk between the two essential phytohormones auxin and cytokinin during reproductive development.

## RESULTS

### HEC1 and SPT act together during gynoecium development

**Fig. 2.**
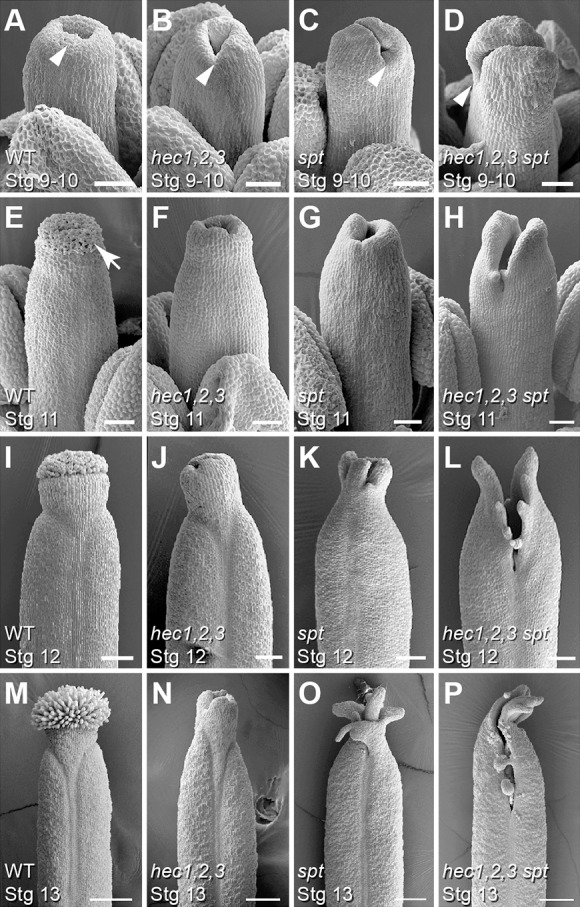
**Development of the *hec1,2,3*, *spt* and *hec1,2,3 spt* mutant gynoecia.** (A-D) Stage 9-10 gynoecia of wild type (A), *hec1,2,3* (B), *spt* (C) and *hec1,2,3 spt* (D) mutant plants. Whereas the gynoecial tube in the medial regions of wild-type plants (A) is extending, *hec1,2,3* (B) and *spt* (C) mutant gynoecia show a retarded growth in this region (arrowheads). This phenotype is enhanced in *hec1,2,3 spt* mutants (D). (E-H) Stage 11 gynoecia of wild type (E), *hec1,2,3* (F), *spt* (G) and *hec1,2,3 spt* (H) mutant plants. Stigmatic papillae (arrow) appear at the top of the wild-type gynoecium (E), but are absent in *hec1,2,3* (F). Carpel fusion defects of the *hec1,2,3 spt* quadruple mutant become more prominent (H). (I-P) Stage 12 (I-L) and stage 13 (M-P) gynoecia of wild type (I,M), *hec1,2,3* (J,N), *spt* (K,O) and *hec1,2,3 spt* (L,P). Note that developing ovules are visible externally in the quadruple mutant (L,P). Scale bars: 50 µm in A-H; 100 µm in I-L; 200 µm in M-P.

We have recently shown that HEC1 physically interacts with SPT *in vivo* to regulate stem cell proliferation in the SAM (Schuster et al., 2014). Because both bHLH transcription factors additionally play important roles during female reproductive development (Gremski et al., 2007; Heisler et al., 2001), we hypothesised that this interaction might also be relevant in the developing gynoecium. During *Arabidopsis* development, *HEC1* and *SPT* are co-expressed in the SAM, early flower primordia and in the carpel (supplementary material Fig. S1) (Gremski et al., 2007; Heisler et al., 2001; Schuster et al., 2014). To further characterise the expression of both transcription factors during carpel and fruit development at high spatio-temporal resolution, we performed β-glucuronidase assays on reporter lines and found that the promoters of *HEC1* and *SPT* exhibit very similar activity patterns (supplementary material Fig. S1). This result, together with the mutant phenotypes reported previously, supported the idea that HEC1 and SPT might functionally interact throughout female reproductive development. To test this interaction genetically, we went on to create the *hec1,2,3 spt* quadruple mutant. Both parents displayed short fruits, with *hec1,2,3* being completely infertile, whereas *spt* sustained moderate fertility but showed unfused carpels at the stylar region (Fig. 1C) (Alvarez and Smyth, 1999; Gremski et al., 2007). The *hec1,2,3 spt* quadruple mutant exhibited a dramatically enhanced phenotype compared with both parents, with carpels being completely unfused at the apical part, up to one third of the entire fruit, illustrating the synergistic activities of these transcription factors (Fig. 1C). To trace the defects of triple and quadruple mutants during early morphogenesis of the gynoecium, we used scanning electron microscopy (SEM) and found that at stage 9-10, *hec1,2,3* triple mutants showed a retarded growth of the gynoecial tube in the medial region compared with wild type, similar to what has been reported for *spt* (Fig. 2A-C) (Alvarez and Smyth, 2002). This phenotype was severely enhanced in the *hec1,2,3 spt* quadruple mutant (Fig. 2D). At later developmental stages, carpel fusion defects of the *hec1,2,3 spt* quadruple mutant became even more prominent (Fig. 2E-P) and remarkably, *hec1,2,3* mutants failed to form any stigmatic papillae at the apex at the onset of stage 11 (Fig. 2E,F). Together, these results confirm that *HEC* and *SPT* genetically interact during reproductive tissue formation and provide direct evidence that *HEC* genes are required for carpel fusion in the developing gynoecium.

### HEC1 regulates auxin biosynthesis and transport in the gynoecium and fruit

**Fig. 3.**
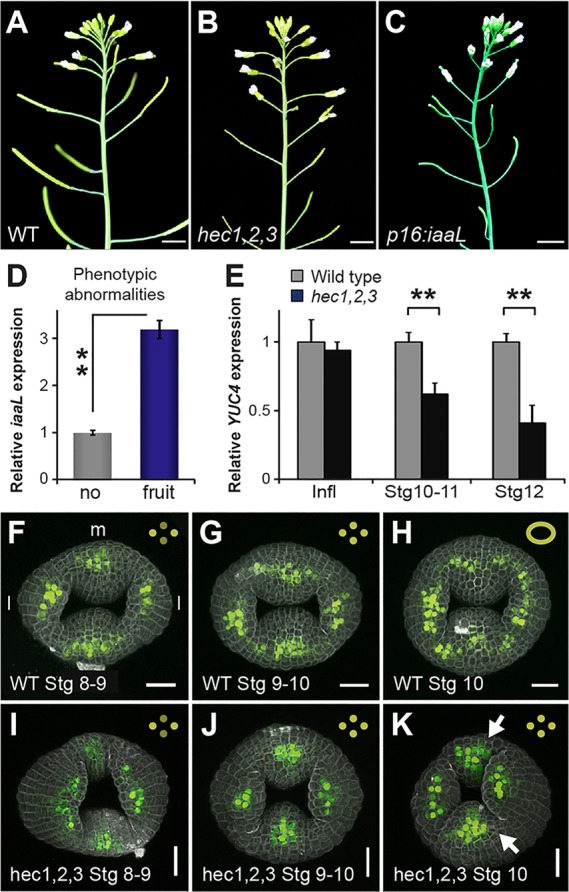
**Loss of *HEC* function leads to impaired auxin signalling.** (A-D) Wild-type (A), *hec1,2,3* (B) and *p16:iaaL* (C) plants. Overexpressing the bacterial *iaaL* gene mimics the *hec1,2,3* fruit phenotype. The overexpression phenotype correlates with the expression level of the *iaaL* transgene; plants with strong *iaaL* expression show distinct fruit phenotypes, whereas weak transgene expression does not cause any phenotypic alterations (D). (E) mRNA expression levels of *YUC4* in dissected inflorescences (Infl) and gynoecia at stage 10-11 and stage 12 of wild type and *hec1,2,3*. **P<0.01. Error bars: s.d. of two (D) or three (E) biological replicates. (F-K) *pDR5:3xYFP-NLS* expression in the developing gynoecium of wild type (F-H) and *hec1,2,3* mutants (I-K) at stage 8-9 (F,I), stage 9-10 (G,J), and stage 10 (H,K). In wild type, four regions of local auxin response are connected to a ring-like structure at the onset of stage 10 (F-H, insets), but *hec1,2,3* mutants fail to establish this radial symmetry (I-K). l: lateral regions; m: medial regions. Arrows in K indicate retarded growth in the medial region. F-K, *n*≥9. Scale bars: 0.5 cm (A-C) and 20 µm (F-K). See also supplementary material Figs S2 and S3.

In a recent study, it was found that SPT and IND cooperate to locally modulate auxin signalling output by directly regulating the expression of *PID* (Girin et al., 2011). A number of observations suggested that *HEC* genes could also be involved in mediating auxin signalling during gynoecium development: First, *hec1,2,3* mutants showed enlarged petals and complete infertility (Fig. 3A,B; supplementary material Fig. S2) (Gremski et al., 2007), traits which have been linked to defects in the regulation of auxin transport, signalling, or synthesis in numerous studies (Cheng et al., 2006; Okada et al., 1991; Varaud et al., 2011). Second, Gremski and colleagues showed that overexpressing any of the *HEC* genes using the *p35S* promoter resulted in plants with *pin*-like inflorescences, very similar to *pin1* and *pid* auxin transport mutants (supplementary material Fig. S2) (Gremski et al., 2007), suggesting that HEC regulators can potently interfere with auxin homeostasis, transport or response. Third, genetic reduction of auxin levels in *yucca 1* (*yuc1*) *yuc2 yuc6* triple mutants and *yuc1 yuc4* double mutants causes fruit phenotypes very similar to *hec1,2,3* (Cheng et al., 2006; Gremski et al., 2007). As the *YUC* genes code for flavin monooxygenases and are central components of the auxin biosynthesis pathway, decreased auxin levels might thus underlie the *hec1,2,3* phenotype.

**Fig. 4.**
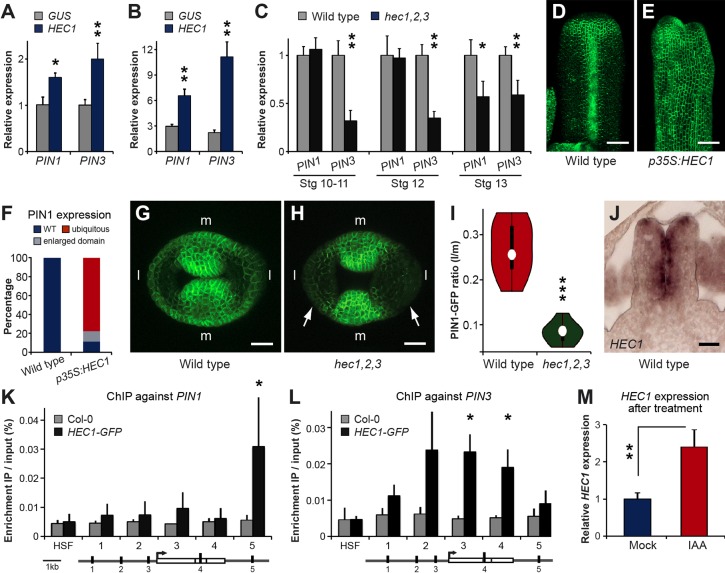
**HEC1 controls *PIN* expression.** (A-B) mRNA expression levels of *PIN1* and *PIN3* in inflorescences of *pAlcA:GUS* and *pAlcA:HEC1* plants after ethanol induction (A) or ethanol induction and auxin (IAA) treatment (B) measured by qRT-PCR. (C) Expression of *PIN1* and *PIN3* in gynoecia of wild type and *hec1,2,3* at multiple developmental stages measured by qRT-PCR. (D-F) *pPIN1:PIN1-GFP* activity in stage 10 fruits of wild type (D) and *p35S:HEC1* (E). In contrast to wild type (D and F; *n*=23), *p35S:HEC1* show ubiquitous PIN1-GFP expression (E and F; *n*=27). (G-I) Reduction of *pPIN1:PIN1-GFP* expression at stage 9-10 in the lateral part (l) of *hec1,2,3* gynoecia (H and I, *n*=9 plants with 3 gynoecia imaged) compared with wild type (G and I, same sample size as *hec1,2,3*). Image analysis revealed a significant difference in the lateral:medial (l/m) PIN1-GFP intensity ratio (***P=3.5×10^−6^, GFP signal threshold=4σ). (J) *HEC1* mRNA expression in wild type stage 8. (K,L) ChIP experiment against *PIN1* (K) and *PIN3* (L) using a stable *p35S:HEC1-GFP* line. *HSF1* served as negative control. (M) *HEC1* mRNA expression in mock and IAA-treated wild-type inflorescences. Error bars: s.d. of three (C,K-M) or four (A,B) biological replicates. *P<0.05; **P<0.01. Scale bars: 50 µm (D,E) and 20 µm (G,H,J). See also supplementary material Fig. S4.

To test the connection between *HEC* genes and the auxin pathway experimentally, we first reduced the pool of active auxin by overexpressing the auxin conjugating enzyme iaaL and observed defects that closely resembled *hec1,2,3* mutant fruit phenotypes (Fig. 3A-D). Building on this result, we analysed expression levels of *YUC* transcripts in wild-type and *hec1,2,3* mutant inflorescences and gynoecia at multiple developmental stages to identify potential regulatory interaction between the HEC factors and *YUC* genes. Whereas the mRNA levels of *YUC1*, *YUC2* and *YUC6* were unaffected, *YUC4* abundance was strongly reduced in *hec1,2,3* throughout gynoecium development (Fig. 3E). Consistently, similar to *HEC1*, *YUC4* is expressed at the apex of wild-type gynoecia (Fig. 4J; supplementary material Fig. S1) (Cheng et al., 2006). The specific effect on *YUC4* expression indicated that the *hec1,2,3* triple mutant phenotype might at least partially be caused by decreased auxin biosynthesis levels.

Recently, IND, another bHLH transcription factor closely related to the HEC factors, has been shown to directly regulate auxin distribution in the fruit (Sorefan et al., 2009). Therefore, we next followed auxin responses during gynoecium and fruit development in wild type and *hec1,2,3* mutants by using the *pDR5:3xYFP-NLS* reporter (Benková et al., 2003) to investigate defects in the spatial organisation of auxin signalling. Wild-type gynoecia showed the previously described characteristic transition from a bilateral symmetry of auxin signalling output with two medial and two lateral foci at stage 8 to a radial symmetry at stage 10, which is required for the radialisation of the style (Fig. 3F-H) (Moubayidin and Østergaard, 2014). In contrast, *hec1,2,3* mutant gynoecia failed to form this ring-like *DR5* expression pattern and retained a pattern with four prominent and isolated auxin signalling foci (Fig. 3I-K). This illustrated that *HEC* gene function is necessary for the auxin radialisation process, and provided evidence that spatially disturbed auxin signalling might underlie the observed carpel fusion, split-style and stigma developmental defects of *hec1,2,3* and *hec1,2,3 spt* mutants. In addition, we observed a strong difference in the *pDR5:3xYFP-NLS* signal between wild type and *hec1,2,3* in the replum of stage 17 fruits, a later stage of reproductive development at which the *hec1,2,3* fruit phenotype became more prominent. In wild type, *DR5:3xYFP-NLS* signal was present in the replum region, but not in the separation layer at the flanks of the replum. A local auxin minimum is required for valve margin specification, and a failure in defining the valve margin separation layer leads to reduced seed dispersal, as observed in *ind* and *spt* mutants (Girin et al., 2011; Sorefan et al., 2009). In contrast to wild type, fruits of *hec1,2,3*, however, did not show consistent YFP signal in the central replum, but rather a moderate signal was found at the valve margin crease (supplementary material Fig. S3). Consistently, the *pHEC1:GUS* reporter indicated that *HEC1* is expressed in the replum and septum of fruits (supplementary material Fig. S3), suggesting that HEC1 might also have a function in regulating auxin distribution or response during post-fertilisation development.

### HEC1 controls PIN1 expression

Our data indicated that HEC1 activity is essential for proper spatio-temporal auxin signalling during gynoecium development. But what are the mechanisms that possibly translate HEC1 activity into specific auxin outputs apart from modulation of biosynthesis? To explore a potential function of HEC1 in the regulation of auxin transport, we performed real-time qRT-PCR analyses on inflorescences and gynoecia at multiple stages of development using the *hec1,2,3* mutant as well as an inducible *HEC1* allele (*p35S:AlcR; AlcA:HEC1*) and analysed the transcriptional response of major components of the polar auxin transport machinery. After overnight induction, *PIN1* and *PIN3* abundance was significantly increased in inflorescences of the *pAlcA:HEC1* line compared with the respective *GUS* control and this effect was further enhanced by auxin co-treatment (Fig. 4A,B). Conversely, *PIN3* expression was strongly reduced in *hec1,2,3* compared with wild type throughout gynoecium development, and lower *PIN1* levels were found from stage 13 (Fig. 4C). Interestingly, we did not observe any changes in the transcript levels of the *PID* and *WAG2* kinases, which facilitate PIN polarisation and are directly regulated by IND, illustrating a specific regulation of different components of the auxin transport machinery by related bHLH transcription factors (supplementary material Fig. S4) (Sorefan et al., 2009). To assess the effect of the HEC regulators on *PIN* expression in the developing gynoecium with cellular resolution, we transformed a constitutive *p35S:HEC1* construct into a stable *pPIN1:PIN1-GFP* reporter line (Heisler et al., 2005) and analysed stage 9/10 gynoecia where morphology was still relatively unaffected. In wild type, the PIN1-GFP fusion protein specifically accumulated in cells at the top of the gynoecium and in the presumptive replum. Gynoecia of *p35S:HEC1* plants instead displayed widespread and ectopic PIN1-GFP expression, consistent with the results of the real-time qRT-PCR analysis (Fig. 4D-F). Conversely, we found a reduction of PIN1-GFP protein abundance in the apical lateral part of stage 9-10 gynoecia from *hec1,2,3* mutants crossed with the *pPIN1:PIN1-GFP* reporter line compared with wild type, whereas medial PIN1-GFP expression was unchanged (Fig. 4G-H). Using quantitative image analysis, we could confirm that the differences in the lateral/medial PIN1-GFP accumulation ratio between wild type and *hec1,2,3* were statistically significant (Fig. 4I). These findings were also consistent with the expression of *HEC1* in lateral spots at the apical part of the early gynoecium (Fig. 4J).

Taken together, our results indicate that HEC activity in the developing gynoecium is necessary and sufficient to drive apical *PIN1* expression. To test the directness of the HEC1-*PIN1/3* regulatory interaction, we next performed chromatin immunoprecipitation (ChIP) experiments followed by qPCR using *p35S:HEC1-GFP* and wild-type control seedlings. In the *HEC1-GFP* line we found a significant enrichment of a fragment downstream of *PIN1* as well as of fragments from the *PIN3* promoter and 3rd intron (Fig. 4K,L). In summary, these results show that HEC1 promotes auxin transport by directly activating the expression of *PIN1* and *PIN3* efflux carriers. Lastly, we wondered whether *HEC1* expression itself might be under control of auxin. To this end, we analysed *HEC1* transcript levels in inflorescences of wild-type plants that had been treated with auxin (50 µM IAA) for 2 h. We found that *HEC1* mRNA expression was elevated (Fig. 4M), demonstrating that *HEC1* is tightly integrated into the auxin signalling network both at the input and output level.

### Dual mode of HEC1 function in SAM maintenance and gynoecium development

**Fig. 5.**
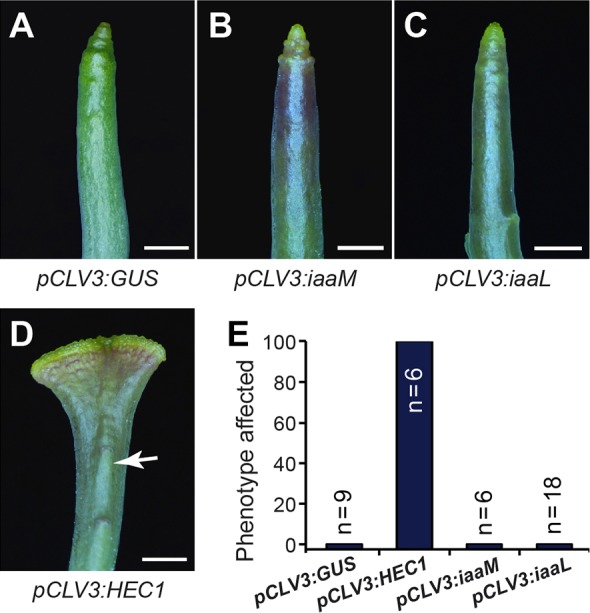
***HEC1*-mediated stem cell over-proliferation is independent of auxin transport and concentration.** (A-D) Transgenic plants expressing *GUS* (A), *iaaM* (B), *iaaL* (C) or *HEC1* (D) under the control of the *CLV3* promoter in a *pin1* mutant background. All *pCLV3:HEC1-pin1* T1 lines showed enlarged meristems, while *pCLV3:GUS-pin1* controls, *pCLV3:iaaM-pin1* and *pCLV3:iaaL-pin1* T1 lines did not (E). Scale bars: 1 mm. See also supplementary material Fig. S5.

Having shown on a regulatory basis that *HEC1* impinges on auxin biosynthesis and transport during gynoecium development, we asked whether this mechanism is also important for the stem cell control activities of *HEC1* in the SAM (Schuster et al., 2014). Because the balance between auxin and cytokinin in the centre of the meristem is essential for stem cell maintenance (Zhao et al., 2010), we tested the effects of HEC1 in a setting with greatly reduced polar auxin transport. Not only should a *pin1* mutant background provide a sensitised environment for testing the effects of elevated or decreased auxin levels in stem cells, but also reveal whether HEC regulators control stem cell behaviour by modulating *PIN1* expression, as in this scenario their effect should be fully supressed. Expression of *HEC1* from the *pCLV3* promoter in a *pin1* mutant background caused massive stem cell over-proliferation, just as in wild type (Fig. 5A,D,E), demonstrating that HEC1 function in shoot stem cells is independent of *PIN1* activity. Consistently, we did not observe obvious changes in PIN1 expression or localisation in SAMs of a *p35S:HEC1* line that carried a *pPIN1:PIN1-GFP* reporter construct and showed a significantly enlarged meristem (supplementary material Fig. S5).

Next, we extended our study and tested if the effects of locally reduced or enhanced auxin levels in general could influence stem cell proliferation in the shoot apex as they do affect morphogenesis of the gynoecium. To this end, we introduced *pCLV3:iaaL* or *pCLV3:iaaM* transgenes, which locally reduced or enhanced auxin content in the stem cell domain, respectively, into the *pin1* mutant background (Romano et al., 1991, 1995). Neither of these constructs provoked an over-proliferation phenotype (Fig. 5B,C,E). Based on these findings, we conclude that the regulation of auxin signalling is likely to be important for the roles of *HEC1* in gynoecium development, while in stem cell regulation *HEC1* acts through a diverse set of transcriptional targets.

### HEC1 and SPT mutants are hypersensitive to cytokinin

**Fig. 6.**
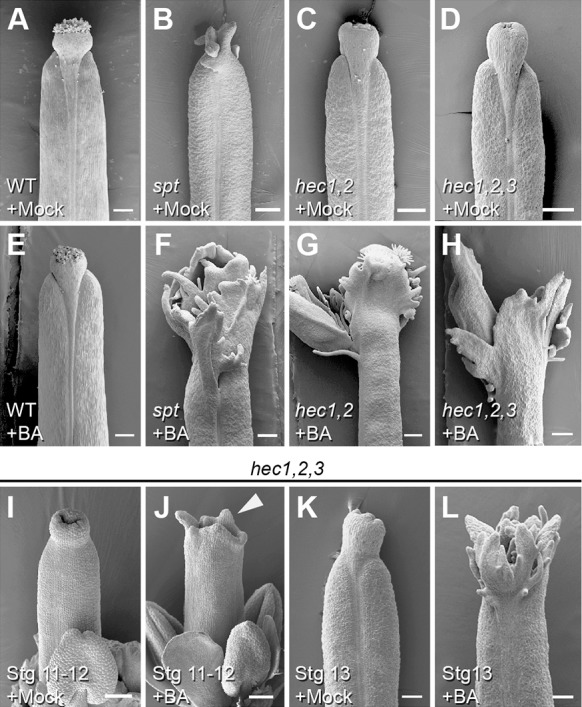
***hec* and *spt* mutants are hypersensitive to cytokinin.** (A-H) Scanning electron microscopy of wild-type (A,E), *spt* (B,F), *hec1,2* (C,G) and *hec1,2,3* (D,H) fruits after mock (A-D) or cytokinin (50 µM BA) (E-H) treatment. Cytokinin treatment of *spt*, *hec1,2* and *hec1,2,3* mutants lead to apically unfused fruits showing ectopic tissue proliferation (F-H), whereas fruits of wild-type plants do not display any phenotypic alterations after treatment compared with mock controls (A,E). SEM images show stage 17b fruits, except panel B (stage 15). (I-L) *hec1,2,3* mutant gynoecia stage 11-12 (I,J) and stage 13 (K,L) after mock (I,K) and cytokinin (J,L) treatment. The arrowhead in J indicates the extensions at the top of the gynoecium. Scale bars: 200 µm (A-H) and 100 µm (I-L). See also supplementary material Fig. S6.

A major function of HEC1 in the context of the SAM is the regulation of cytokinin response by activating the expression of several type-A ARRs (Schuster et al., 2014). Interestingly, recent observations indicate that the phytohormone cytokinin is also important for ovule development, gynoecium as well as fruit patterning and morphogenesis (Bartrina et al., 2011; Marsch-Martínez et al., 2012a,b). We therefore tested whether *HEC* genes play a role in regulating cytokinin signalling in the gynoecium and fruit. To this end, we used a pharmacological approach and treated developing flowers of *hec1,2*, *hec1,2,3* and *spt* mutants with cytokinin at levels that do not cause any phenotypic alterations in wild type (50 µM BA). While fruits of wild-type plants did not show any morphological changes (Fig. 6A,E), both *hec* and *spt* mutants were hypersensitive to cytokinin: the fruit was apically unfused and displayed extensive tissue proliferation at the top (Fig. 6B-D versus F-H). This phenotype was already present in gynoecia of cytokinin treated *hec1,2,3* mutants at earlier stages of development (Fig. 6I-L). It is important to mention that extensive cytokinin treatment of wild-type plants led to massive over-proliferation at the external medial region along the entire fruit, but never led to unfused carpels and apically restricted tissue proliferation as observed in *hec* and *spt* mutants (supplementary material Fig. S6) (Marsch-Martínez et al., 2012a). Taken together, the over-proliferation phenotype of cytokinin-treated *hec* and *spt* mutants points towards a function of the HEC1-SPT module in negatively modulating cytokinin signalling during gynoecium development, in line with the reported activation of negative cytokinin signalling components by HEC1.

## DISCUSSION

### HEC1 and SPT interact to control gynoecium development

Our phenotypic analysis of the *hec1,2,3 spt* quadruple mutant showed that *HEC1* and *SPT* genetically interact during gynoecium development. Combined with the significant overlap of their spatio-temporal expression pattern and the previously reported protein interaction both in yeast and *in planta* (Gremski et al., 2007; Schuster et al., 2014), this clearly points towards a role for the HEC1-SPT complex during female reproductive development. It should be pointed out that *hec* and *spt* mutants show shared as well as clearly distinct phenotypes. Whereas both *hec* and *spt* mutants exhibit short fruits and reduced fertility as a result of disturbed gynoecium development (Alvarez and Smyth, 1999; Gremski et al., 2007; Heisler et al., 2001), the most obvious difference is that *hec1,2,3* plants, in contrast to *spt* mutants, have only minor defects related to gynoecium fusion. The combination of *hec1,2,3* and *spt*, however, severely enhanced this aspect of the *spt* mutant phenotype. A comparable phenomenon was observed in *alcatraz-1* (*alc-1*) *spt-2* double mutants. Here, similar to *hec1,2,3*, *alc-1* alone did not show any carpel fusion defects, but the double mutant exhibited a substantial enhancement of this particular phenotype (Groszmann et al., 2011). One possible scenario to explain this would involve the functional redundancy of *HEC* genes with other bHLH transcription factors, including *SPT*, during female reproductive development. Interestingly, we previously showed that in the context of the shoot apical meristem, HEC function is the restricting component of the HEC1-SPT module, but during gynoecium patterning, SPT seems to be limiting. It will be important to analyse the role of *ALC* in this regulatory system in future studies. As the margins of the two fused carpels display meristematic characteristics, the role of *HEC1* in carpel fusion presented in this study fits well with its function as a regulator of cell proliferation in the SAM. Taken together, we have demonstrated the importance of the *HEC-SPT* module in the developing gynoecium and provide evidence for a yet unknown role of the *HEC* genes during carpel fusion.

### HEC1 regulates phytohormone responses in the developing gynoecium

Phytohormones are known to play key roles during flower and fruit development. Whereas the importance of auxin and gibberellin is well established, the role of cytokinin function in reproductive tissue development is less well understood (Arnaud et al., 2010; Daviere and Achard, 2013; Østergaard, 2009). Recent studies demonstrate that cytokinin promotes cell proliferation in early reproductive tract development and regulates valve margin morphogenesis at later stages (Bartrina et al., 2011; Marsch-Martínez et al., 2012a,b). Here, we found that both *hec* and *spt* mutants are hypersensitive to cytokinin treatment. As HEC1/2/3 can activate type-A *ARR* genes, which are negative regulators of cytokinin signalling, this observation suggests that HEC transcription factors function by restricting cytokinin responses during gynoecium development, as supported by the massive tissue proliferation at the apex of the gynoecium in *hec1,2,3* upon cytokinin treatment.

**Fig. 7.**
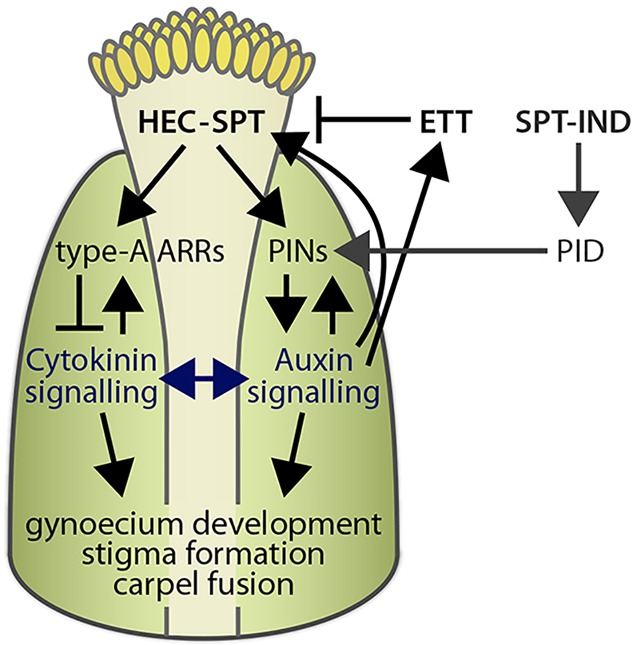
**Hypothetical model of *HEC* gene function during gynoecium development.** HEC1 interacts with SPT to control carpel fusion, and both transcription factors buffer auxin and cytokinin signals during gynoecium development. This might involve type-A ARRs, which antagonise cytokinin function. HEC1 stimulates auxin biosynthesis and directly activates the expression of *PIN1* and *PIN3* auxin efflux transporters and thus ultimately regulates auxin distribution during early stages of gynoecium development. Interestingly, the SPT-IND complex binds to the promoter of the *PID* gene that modulates PIN polarisation. This highlights how combinatorial effects of related bHLH transcription factors regulate distinct components of the auxin signalling machinery. Finally, HEC1 itself is tightly integrated into the auxin signalling network, and its spatial expression seems to be partly controlled by auxin-dependent activation and ETT mediated repression. Cross-talk between auxin and cytokinin pathways is an important feature of shoot meristem control and might also play a role in the developing gynoecium and fruit.

In addition to the regulation of cytokinin responses, we also found that HEC1 modulates auxin biosynthesis and distribution in the gynoecium by activating the expression of *YUC4*, as well as *PIN1* and *PIN3* genes, respectively. Interestingly, a previous study demonstrated that SPT interacts with IND to regulate the expression of *PID* and thus ultimately controls polar localisation of PIN proteins (Fig. 7) (Girin et al., 2011). This nicely demonstrates how related bHLH transcription factors can control different components of the same signalling pathway. We propose that the lack of PIN1 expression in the apical lateral part of early gynoecia observed in *hec1,2,3* mutants might prevent the establishment of the auxin radial symmetry, which is required for the radialisation of the style (Moubayidin and Østergaard, 2014). In addition to the role of *HEC* genes in regulating auxin signalling during gynoecium development, we also found evidence for an auxin-mediated function of HECs during later stages of female reproductive development. Future experiments should further analyse the *in vivo* relevance of the auxin-HEC regulatory interaction. This could be done by artificially expressing the auxin synthesis gene *iaaM* under the control of the *HEC1* promoter and searching for a *hec1,2,3* mutant fruit phenotype rescue, analogous to previous work done on *yuc1,2,6* (Cheng et al., 2006). Interestingly, in contrast to the regulation of cytokinin signalling, which represents a common feature of HEC function in both SAM and gynoecium, the control of auxin distribution seems to be specific for reproductive development.

Cross-talk between auxin and cytokinin is important for the control of both root and shoot stem cell systems, and has also been proposed to play a role in gynoecium morphogenesis (Marsch-Martínez et al., 2012b; Müller and Sheen, 2008; Zhao et al., 2010). Besides the apical-basal gradient of auxin with high levels of auxin at the top of the gynoecium and low levels at the bottom (Nemhauser et al., 2000), a reverse gradient could exist for cytokinin with a maximum concentration at the basal and a minimum concentration at the apical end (Østergaard, 2009; Sundberg and Østergaard, 2009). Because HEC1 can impinge on both auxin and cytokinin signalling, it needs to be further elucidated whether HEC1 functions as a central hub to balance the local response ratio between both hormones.

In summary, based on the results presented in this study and previous work, we suggest the following working model (Fig. 7): HEC1/2/3 transcription factors control gynoecium development in conjunction with SPT by balancing phytohormone responses, most notably auxin and cytokinin. First, these factors act together, at least in part, by modulating cytokinin action, potentially through the activation of type-A *ARR* genes. Second, HEC1/2/3 control *YUC4* and *PIN* expression and thus ultimately local auxin signalling; we propose that this function is important for gynoecium development, but might also play a role in the development of the fruit. Strikingly, HEC genes themselves are tightly integrated into the auxin signalling network: auxin stimulates the expression of *HEC1*, but also limits HEC and SPT activity through ETT function (Gremski et al., 2007; Heisler et al., 2001). The specific mode of each hormone action influenced by HEC transcription factors needs to be elucidated in future studies.

## MATERIALS AND METHODS

### Plant material and treatments

Plants of Columbia background were grown at 23°C in long days. Ethanol vapour inductions were performed overnight by placing a tip-box filled with 95% ethanol into the plant tray. For inductions with IAA, inflorescences were incubated for 2 h in ½ MS medium containing 50 µM IAA and 0.015% Silwet L-77; 0.1% dimethylsulfoxide (DMSO). 0.015% Silwet L-77 in ½ MS was used as control. Cytokinin treatments were performed by spraying 50 µM 6-benzyladenine (BA) once a week on inflorescences during a 3-week period after bolting.

The *spt* allele corresponds to *spt-12* (Ichihashi et al., 2010), the *pSPT:GUS* reporter line is pSPT:6253-GUS (Groszmann et al., 2010) and the *hec1,2,3* triple mutant was previously described (Schuster et al., 2014).

### Transgenes

The *HEC1* coding sequence was amplified using Gateway tailed primers and cloned into pGEM-T Easy (Promega) for sequencing. For generating constitutive overexpression constructs it was then recombined into pDONR221 using Gateway Technology (Invitrogen) and further recombined into pGREEN II destination vectors carrying tissue-specific promoters. The same procedures were used for making constructs of *GUS* control, *iaaL* and *iaaM* expression. The ethanol-inducible *HEC1* line and the *p16* promoter are described in Schuster et al. (2014). To assess auxin signalling activity, the *DR5* reporter driving the expression of *3xYFP-NLS* was transformed into wild type and *hec1,2,3*. For monitoring PIN1 expression upon alterations of *HEC1* activity, *p35S:HEC1* was transformed into a stable *pPIN1:PIN1-GFP* line [provided by Marcus Heisler (Heisler et al., 2005)], and *hec1,2,3* triple mutants were crossed with the same PIN1 reporter line.

### Quantitative real-time RT-PCR

qRT-PCR was performed on dissected inflorescence apices and on gynoecia at multiple developmental stages. Tissue was collected in microcentrifuge tubes floating on liquid nitrogen. Twenty plant samples were pooled for each replicate, and RNA was prepared using the RNeasy Plant Mini Kit (Qiagen). After DNase treatment, cDNA was prepared from 1 µg total RNA using the RevertAid First Strand cDNA Synthesis Kit (Fermentas). Quantitative real-time RT-PCR was carried out using SYBR Green and amplification of *TUBULIN* served as control. Sequences for all primers are listed in supplementary material Table S1.

### Chromatin immunoprecipitation (ChIP)

Chromatin immunoprecipitation was performed according to (Gendrel et al., 2005) with minor modifications. Col-0 and *p35S:HEC1-GFP* seedlings were harvested 12 days after germination. Overnight immunoprecipitation was performed using GFP-trap (Chromotek) and DNA isolation was conducted using MinElute Reaction Cleanup Kit (Qiagen). For each individual biological replicate, technical duplicates were obtained by splitting the samples after sonication, and by processing them separately in the subsequent steps.

### Microscopy

Confocal laser scanning microscopy, scanning electron microscopy and GUS staining were performed in accordance with standard protocols. The lateral:medial expression ratio of the *pPIN1:PIN1-GFP* reporter in gynoecia of wild type and *hec1,2,3* mutant was determined by thresholding the GFP signal intensity to the tissue background intensity of the same image. The threshold was determined by the mean of the background intensity plus four standard deviations.

### Statistical analysis

For statistical analysis, data was first tested for normality using the Shapiro–Wilk test. Then, means were compared pair-wise using either Welch's *t*-test or the Wilcoxon rank sum test. All calculations were performed in R.
